# Lacunar Stroke Lesion Extent and Location and White Matter Hyperintensities Evolution 1 Year Post-lacunar Stroke

**DOI:** 10.3389/fneur.2021.640498

**Published:** 2021-03-05

**Authors:** Maria del C. Valdés Hernández, Tara Grimsley-Moore, Eleni Sakka, Michael J. Thrippleton, Francesca M. Chappell, Paul A. Armitage, Stephen Makin, Joanna M. Wardlaw

**Affiliations:** ^1^Centre for Clinical Brain Sciences, University of Edinburgh, Edinburgh, United Kingdom; ^2^College of Medicine and Veterinary Medicine, University of Edinburgh, Edinburgh, United Kingdom; ^3^Academic Unit of Radiology, University of Sheffield, Sheffield, United Kingdom; ^4^Centre for Rural Health, University of Aberdeen, Aberdeen, United Kingdom

**Keywords:** recent small subcortical infarct, lacunar, stroke, white matter hyperintensities, vascular risk factors, lacunes of presumed vascular origin

## Abstract

Lacunar strokes are a common type of ischemic stroke. They are associated with long-term disability, but the factors affecting the dynamic of the infarcted lesion and the brain imaging features associated with them, reflective of small vessel disease (SVD) severity, are still largely unknown. We investigated whether the distribution, volume and 1-year evolution of white matter hyperintensities (WMH), one of these SVD features, relate to the extent and location of these infarcts, accounting for vascular risk factors. We used imaging and clinical data from all patients [*n* = 118, mean age 64.9 (SD 11.75) years old] who presented to a regional hospital with a lacunar stroke syndrome within the years 2010 and 2013 and consented to participate in a study of stroke mechanisms. All patients had a brain MRI scan at presentation, and 88 had another scan 12 months after. Acute lesions (i.e., recent small subcortical infarcts, RSSI) were identified in 79 patients and lacunes in 77. Number of lacunes was associated with baseline WMH volume (B = 0.370, SE = 0.0939, *P* = 0.000174). RSSI volume was not associated with baseline WMH volume (B = 3.250, SE = 2.117, *P* = 0.129), but predicted WMH volume change (B = 2.944, SE = 0.913, *P* = 0.00184). RSSI location was associated with the spatial distribution of WMH and the pattern of 1-year WMH evolution. Patients with the RSSI in the centrum semiovale (*n* = 33) had significantly higher baseline volumes of WMH, recent and old infarcts, than patients with the RSSI located elsewhere [median 33.69, IQR (14.37 50.87) ml, 0.001 ≤ *P* ≤ 0.044]. But patients with the RSSI in the internal/external capsule/lentiform nucleus experienced higher increase of WMH volume after a year [*n* = 21, median (IQR) from 18 (11.70 31.54) ml to 27.41 (15.84 40.45) ml]. Voxel-wise analyses of WMH distribution in patients grouped per RSSI location revealed group differences increased in the presence of vascular risk factors, especially hypertension and recent or current smoking habit. In our sample of patients presenting to the clinic with lacunar strokes, lacunar strokes extent influenced WMH volume fate; and RSSI location and WMH spatial distribution and dynamics were intertwined, with differential patterns emerging in the presence of vascular risk factors. These results, if confirmed in wider samples, open potential avenues in stroke rehabilitation to be explored further.

## Introduction

Small subcortical ischemic strokes make up 20–30% of all ischemic strokes (1). Specifically lacunar ischemic strokes occur within the deep white matter, basal ganglia, pons, or brainstem, as result of disease in a small perforating arteriole (2), consistent with a lacunar clinical syndrome (3), and are a feature of cerebral small vessel disease (SVD). These strokes cause symptoms if affecting the motor and sensory pathways (4), and there is a growing interest in the predictors and impact of their long-term fate (5). Despite their small size, they have been associated with long-term disability, leading to a range of physical impairments like reduced mobility (6), gait, and balance impairments (7).

White matter hyperintensities (WMH) are also a feature of SVD identified as regions of increased signal intensity observed in T2-weighted and fluid-attenuated inversion recovery (FLAIR) magnetic resonance imaging (MRI) brain scans. Similar to lacunar infarcts, WMH are associated with increasing age, smoking and hypertension (8–11). There is evidence that after stroke, WMH not only increase but can also regress (12, 13). High WMH burden alone has been identified as a significant risk factor for subsequent stroke, independent of other stroke risk factors (14, 15), with consequent increased risk of mortality attributable to stroke (16). They are related with lacunar strokes, but the nature of this relation is not well-understood. Without enough longitudinal data and a detailed analysis of the extent and location of the stroke in relation to the anatomical distribution of the WMH, it is difficult to elucidate the role of one with respect to the other. For example, WMH progression has been associated with lacunar infarct occurrence, particularly within the first 2 years (17). Conversely, some studies have indicated that WMH are secondary to lacunar infarcts, as lacunar infarcts affect the integrity of white matter tracts (18). A study found that acute lacunar infarcts tended to occur in the cerebral hemisphere with relatively more severe WMH, hence implying that patients with asymmetric WMH may be vulnerable to this type of infarct (19) and that high WMH burden may be an indicator of subsequent lacunar stroke occurrence.

We conduct an exploratory study to investigate the influence of the extent and location of recent small subcortical infarcts (RSSI) and lacunes on WMH volume, spatial distribution, and evolution pattern over a 1-year period in patients identified having had a stroke of type lacunar, accounting for vascular risk factors and age at the time of the stroke; with a specific interest on whether RSSI location can be associated with certain pattern of lesion changes 1 year later. Due to the exploratory nature of our study, we can only hypothesize that the extent and location of RSSI and lacunes will be associated with the spatial distribution of WMH and their progression at 1 year.

### Definitions

Lacunar stroke syndrome—Clinically diagnosed, as pure motor, sensory, sensori-motor stroke, or ataxic hemiparesis, with fast development of clinical symptoms lasting more than 24 h with no apparent cause other than that of vascular origin (20). Neuroimaging examination may or may not show a diffusion-weighted imaging (DWI)—positive lesion in the territory of a perforating arteriole, but should discard other pathologies with similar symptoms (e.g., brain tumor).

Recent Small Subcortical Infarcts (RSSI)—Neuroimaging evidence of recent infarction in the territory of one perforating arteriole, seen as a hyperintense lesion, generally <20 mm diameter in the axial plane, with features or clinical symptoms consistent with a lesion occurring in the previous few weeks (3). As the sample analyzed only involves patients with a lacunar stroke syndrome, we refer to RSSI as the neuroradiological evidence of the lacunar stroke, confirmed by inspection of the diffusion-weighted image (DWI).

Lacunes (of presumed vascular origin)—Neuroimaging feature identified as round or ovoid, subcortical, fluid-filled cavity, with signal similar to CSF, and diameter between 3 and 15 mm, consistent with a previous RSSI or hemorrhage in the territory of one perforating arteriole (3).

## Materials and Methods

### Subjects

We analyzed prospectively collected imaging and clinical data from all patients presenting with an acute stroke to a regional hospital within the years 2010 to 2013, who consented to participate in a study of stroke pathophysiology (21). From the 264 patients recruited for the study, 118 (67 males, 51 females) had a lacunar stroke clinical syndrome according to the Oxfordshire Community Stroke Project Classification (20). The lacunar stroke (3) diagnosis was reached by consensus by an expert team of stroke physicians and neuroradiologists after examining clinical and neuroradiological evidence. Patients were aged 18 years and over, and were excluded from the primary study that provided data for this analysis if they lacked the capacity to consent or had a medical condition that made follow-up clinical assessment unlikely or impossible, magnetic resonance imaging (MRI) examination suggested an alternative diagnosis (e.g., multiple sclerosis or cancer), did not tolerate an MRI scan or suffered severe renal impairments. The flow chart of the recruitment process in the primary study is published in Heye et al. (22). Written consent was obtained from all patients on protocols approved by the Lothian Ethics of Medical Research Committee (REC 09/81101/54) and NHS Lothian R+D Office (2009/W/NEU/14), on the 29th of October 2009. Full recruitment assessment can be found in these previously published articles (21, 22).

### Clinical Data

The following vascular risk factors, collected at diagnosis, were selected based on relevance and previous stroke research (21). These were physician-diagnosed hypertension (yes, no), hyperlipidemia (yes, no), smoker status [current, recent (i.e., <1 year), non-smoker, ex-smoker (i.e., more than 1 year)] and diabetic status (yes or no). Diabetes type (i.e., one or two) was not considered.

### MRI Acquisition

All MRI scans were acquired using a 1.5T GE Signa Horizon HDxt clinical scanner (General Electric, Milwaukee, WI, USA) operating in research mode and using a self-shielding gradient set with maximum gradient of 33 mT/m and an 8-channel phased-array head coil. Diffusion weighted imaging, fluid attenuation inversion recovery (FLAIR), T2-, T1-, and T2^*^-weighted MRI, were acquired soon after presenting to hospital with acute stroke symptoms and 1 year after with identical protocols as per (22). For processing, all images (i.e., acquired at both time points) were mapped to a common space.

### Image Analysis

#### Stroke Subtype and Lesion Location

The primary study recorded the location, type, distribution and size of all infarcts, new and old and their progression (i.e., if cavitated, disappeared, increased, or decreased in size) (5) using validated scoring (23). We used the number (i.e., count) and location of the recent small subcortical infarcts (RSSI) and lacunes (24, 25) in the following anatomical regions: internal and external capsule/lentiform nucleus, internal border zone, centrum semiovale, thalamus, brainstem, cerebellum and optical radiation (23) (proforma available from www.ed.ac.uk/edinburgh-imaging/analysis-tools/stroke).

#### WMH and Stroke Lesion Volumes

Stroke lesions (old and recent), and intracranial volume (ICV) for each patient were segmented independently at both time-points using a threshold-based semi-automatic segmentation combined with a region-growing algorithm (26), on the FLAIR and T2^*^-weighted images, respectively. WMH and brain tissues were also segmented independently at both time-points using a multispectral approach as described in Valdés Hernández et al. (2015). All data was obtained by request from the primary study database. Briefly, the ICV, defined as contents within the inner skull table including brain tissue, CSF, veins, and dura, and limited inferiorly by the tip of the odontoid peg at the foramen magnum, was extracted using the Object Extraction Tool in Analyze 11.0, followed by manual editing using the Region of Interest Tool of the same software. The later was also used to delineate stroke lesion boundaries. WMH volumes were segmented by thresholding a color fusion of co-registered structural sequences mapped in the red-green color space using MCMxxxVI (www.sourceforge.net/projects/bric1936) and subdivided in regions of intense and less intense signal as per Valdés Hernández et al. (2015). For statistical analysis, volumes, obtained from the binary masks, were calculated as a percentage in the ICV (%ICV). Volumetric measurements were recorded at baseline and 1-year follow up. WMH evolution was determined by combining baseline and follow-up WMH binary masks.

#### Voxel-Based Analysis

All T1-weighted images were semi-rigidly mapped in the standard space (27) using a common age-relevant brain template (28), and non-linear registration (http://sourceforge.net/projects/niftyreg/) through TractoR (http://www.tractor-mri.org.uk/diffusion-processing). The transformation matrix was applied to the WMH binary masks. These were, then, concatenated in a 4D array. Two 4D arrays of WMH were generated: one that used all baseline images (*n* = 118) and other that used all 1-year follow-up images (*n* = 88) combined with the baseline images to represent WMH evolution. The former was used to (1) Investigate in which voxels the WMH of patients with the RSSI/lacunes in a certain location differ from the equivalent voxels in brains of patients with the RSSI/lacunes located elsewhere; and (2) Investigate in which voxels the WMH of patients with the RSSI/lacunes in a certain location and with certain vascular risk factor (e.g., hypertensive patients with the RSSI in the centrum semiovale, current smokers with the RSSI in the basal ganglia, etc.) differ from the equivalent voxels in brains of patients with the RSSI/lacunes elsewhere who did not have such risk factor. The 4D WMH array of WMH evolution was used to investigate whether the WMH progression/regression in a voxel relates to RSSI location.

### Statistical Analysis

Using MATLAB R2019b and Statistical Package for Social Science (SPSS version 25), we performed analysis of frequencies of RSSI and lacunes per anatomical region.

We used linear regression models to investigate association between RSSI volume/number of lacunes and WMH volume at baseline. We used the Kruskal-Wallis test to investigate WMH volumes overt (i.e., intense) and subtle (i.e., less intense) in relation to RSSI lesion location and lacunes. We inspected histograms for normality. A non-parametric Levene's test was used to verify the equality of variances in the samples (homogeneity of variance) (*P* > 0.05) (29, 30).

We used ANCOVA to investigate if RSSI lesion size (i.e., RSSI volume at baseline), location and number of lacunes predicted the WMH volume change in the sample. All models accounted for age. All volumes were adjusted by head size and expressed as percentage in ICV. Models were repeated accounting also for vascular risk factors and baseline volume of old ischemic stroke lesions. All analyses were done twice: (1) considering only the location of the primary RSSI lesion cluster and (2) recoding the RSSI location to account for multiple RSSI clusters (i.e., observed in six patients).

We performed voxel-based statistical comparisons of total WMH maps (i.e., less intense and intense together) per lesion location using the Kruskal-Wallis test, and corrected our results for multiple comparisons using false discovery rate [code available and documented in (31)]. We also implemented a voxel-wise regression model to explore whether the RSSI location was associated with the WMH evolution pattern at 1-year, using a machine-learning approach. This used the MATLAB function “fitrlinear” to fit a regularized Support Vector Machine regression model with a ridge penalty type optimized through a stochastic gradient descent approach for accuracy. This model was selected due to the high-dimensionality and sparsity of the predictor data. Covariates were age and RSSI location (i.e., the later coded as per the proforma previously mentioned). The regularization term strength was set at 1/47.

## Results

### Sample Characteristics

*Demographics*—From the 118 patients with baseline data, 88 had complete brain MRI acquired at 1 year (Table 1). The 30 patients not followed-up either declined further MRI scan or were too unwell. The mean age of the sample at baseline was 64.9 years old (SD 11.75). The proportion of males to females in the two time points was similar with marginally more males in each. The number of males (M) with RSSI in the two locations with the highest frequency of occurrence (i.e., internal/external capsule/lentiform nucleus and centrum semiovale) was almost double the number of females (F) (13M/7F and 20M/13F, respectively) (see Supplementary Figure 1).

**Table 1:** Sample characteristics (*n* refers to number of patients).

**Variable type**	**Baseline measurements**		**1-year follow-up measurements (n = 88)**
	**Baseline sample (*n* = 118)**	**Subsample that provided measurements at 1-year follow-up (*n* = 88)**	
**Age (years) [mean(SD)]**	64.93 (11.75)	64.79 (10.87)	
**Gender [*****n*** **(%)]**			
Male	67 (57)	52 (59)	52 (59)
Female	51 (43)	36 (41)	36 (41)
**Brain measurements**			
ICV (ml) [mean (SD)]	1469.20 (139.82)	1469.04 (140.87)	1469.04 (140.87)
Old Stroke Lesion Volume (%ICV) [median (IQR)]	0.084 (0.038–0.197) (*n* = 42)	0.093 (0.042–0.239) (*n* = 29)	0.11 (0.050–0.227) (*n* = 26)
Index Stroke Lesion Volume (%ICV) [median (IQR)]	0.075 (0.048–0.115) (*n* = 77)	0.076 (0.054–0.125) (*n* = 61)	0.042 (0.026–0.083) (*n* = 56)
Total WMH Volume (%ICV) [median (IQR)]	0.944 (0.310–2.466) (*n* = 117)	1.037 (0.351–2.448) (*n* = 88)	1.094 (0.452–2.666) (*n* = 88)
Volume of severe (i.e., intense) WMH (%ICV) [median (IQR)]	0.216 (0.104–0.689) (*n* = 117)	0.216 (0.116–0.623) (*n* = 88)	0.303 (0.128–0.729) (*n* = 87)
Volume of subtle (i.e., less intense) WMH (%ICV) [median (IQR)]	0.689 (0.197–1.767) (*n* = 116)	0.759 (0.267–0.179) (*n* = 87)	0.785 (0.334−1.644) (*n* = 87)
**Medical history acquired at baseline [*****n*** **(%)]**			
Diabetes	12 (10.2)	8 (9.0)	
Hypertension	82 (69.5)	65 (73.9)	
Hyperlipidemia	73 (61.9)	56 (63.6)	
Current smoker	46 (39.0)	33 (37.5)	
Recent smoker	5 (4.2)	3 (3.4)	
Ex-smoker	31 (26.3)	22 (25.0)	
Non-smoker	35 (29.7)	30 (34.1)	
**Small subcortical infarcts in the internal/external capsule/lentiform nucleus [No. features]**			
RSSI left hemisphere	9	8	
RSSI right hemisphere	13	9	
Lacunes left hemisphere	15	13	
Lacunes right hemisphere	17	14	
**Small subcortical infarcts in the internal border zone [No. features]**			
RSSI left hemisphere	0	0	
RSSI right hemisphere	0	0	
Lacunes left hemisphere	1	1	
Lacunes right hemisphere	1	0	
**Small subcortical infarcts in the centrum semiovale [No. features]**			
RSSI left hemisphere	16	13	
RSSI right hemisphere	18	15	
Lacunes left hemisphere	14	10	
Lacunes right hemisphere	18	10	
**Small subcortical infarcts in the thalami [No. features]**			
RSSI left hemisphere	6	6	
RSSI right hemisphere	7	6	
Lacunes left hemisphere	4	2	
Lacunes right hemisphere	3	3	
**Small subcortical infarcts in the brainstem [No. features]**			
RSSI left hemisphere	3	3	
RSSI right hemisphere	7	3	
Lacunes left hemisphere	2	2	
Lacunes right hemisphere	3	2	
**Small subcortical infarcts in the cerebellum [No. features]**			
RSSI left hemisphere	0	0	
RSSI right hemisphere	0	0	
Lacunes left hemisphere	2	1	
Lacunes right hemisphere	1	0	
**Small subcortical infarcts in the optical radiation [No. features]**			
RSSI left hemisphere	2	2	
RSSI right hemisphere	0	0	
Lacunes left hemisphere	5	5	
Lacunes right hemisphere	2	2	

*Brain measurements are calculated considering only the subsample with volume greater than zero and expressed as percentage in intracranial volume (ICV) (i.e., adjusted for head size). When the sample size differs from the number of patients that provided measurements at each time point, this is indicated as (n = sample size). Baseline measurements are given for both: the baseline sample (n = 118) and the subsample that provided measurements at follow-up (n = 88)*.

*Brain measurements—*Median RSSI volume at baseline was greater than at 1-year, while the volume of WMH was slightly higher at follow-up than at baseline (Table 1). Baseline lesion volumes of the subsample followed-up at 1 year were not different from those of the whole sample (Table 1).

**Figure 1:**
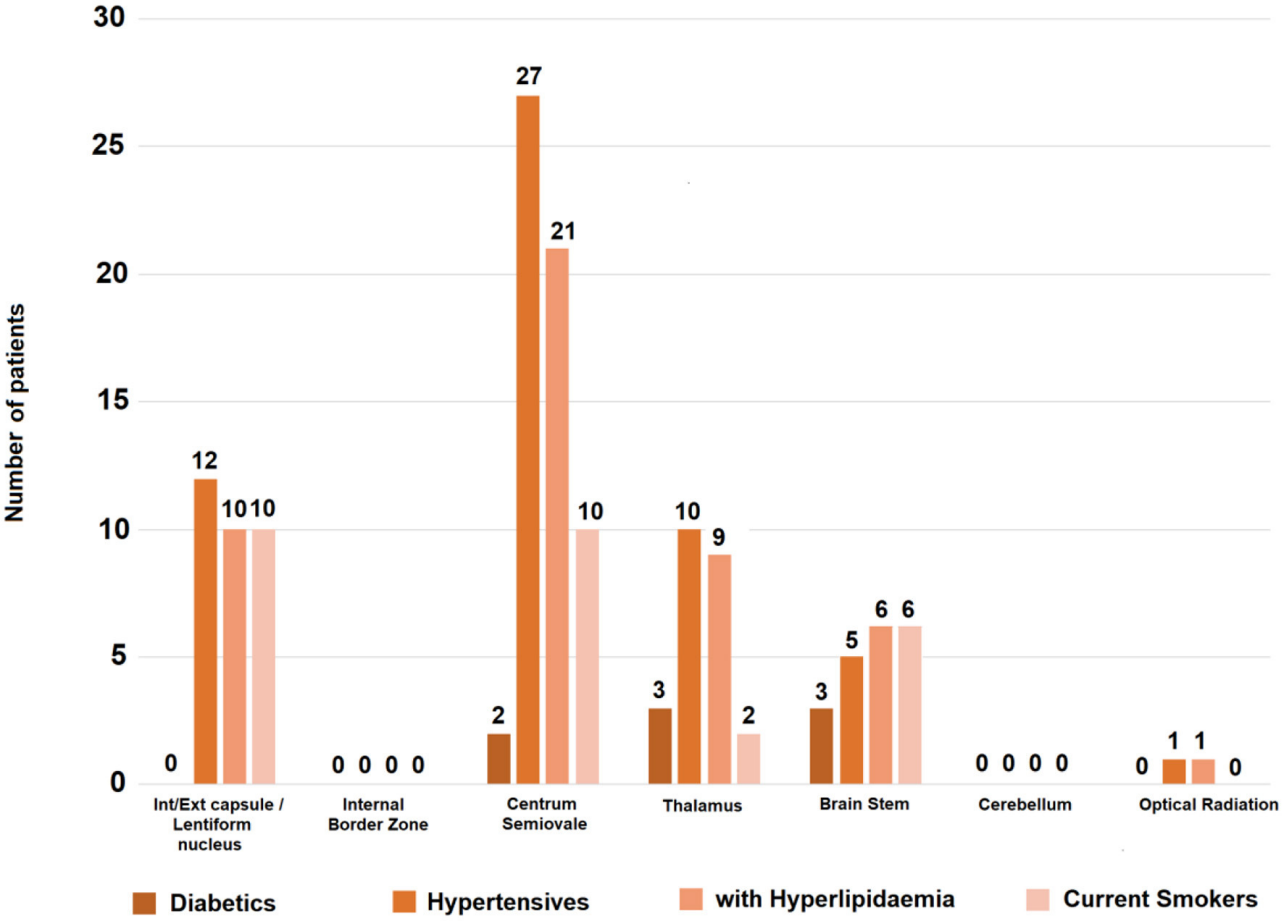
Vascular risk factors per RSSI location in the sample at baseline. The vertical axis indicates number of patients with the risk factor specified. From the patients with RSSI in the internal/external capsule/lentiform nucleus, 57% (12/21) were hypertensive, 48% (10/21) had hyperlipidemia and the same proportion (i.e., 10/21) were current smokers. From those with the RSSI in the centrum semiovale, 81.8% (27/33) were hypertensive, 63.6% (21/33) had hyperlipidemia and 30.3% (10/33) were current smokers. Proportions were higher in patients with the RSSI in brainstem (*n* = 10) and thalamus (*n* = 13) but samples were small.

*Vascular risk factors*−10% of patients were diabetics, 70% hypertensive, 62% had hyperlipidemia and 43% were smokers (Table 1). Figure 1 and Supplementary Figure 1 show the vascular risk factors of patients grouped per RSSI location.

*RSSI and lacunes*—RSSI were identified in 79/188 patients. One patient had two RSSI clusters in the centrum semiovale in the same cerebral hemisphere, and one patient had a bilateral RSSI in the internal/external capsule. Three patients had additional cortical DWI-positive lesions. One patient had an RSSI cluster in the internal/external capsule and other in the brainstem. The rest had only one RSSI cluster. Lacunes were identified in 77/118 patients, multiple lacunes in 20/118 patients. The highest frequency of RSSI and lacunes was in the internal and external capsule/lentiform nucleus and centrum semiovale (Tables 1–3). The RSSI and lacunes distribution in left and right hemispheres was fairly even.

**Table 2:** Average (i.e., median) and interquartile range values [i.e., median (Q1 Q3)] of lesion volume (ml) per RSSI location in the baseline and follow-up samples.

**RSSI location**	**Time point**	**Number of patients**	**Intense (severe) WMH volume (ml)**	**Less intense (subtle) WMH volume (ml)**	**Total (i.e., intense and less intense) WMH volume (ml)**	**Index stroke lesion (RSSI) volume (ml)**	**Old ischemic stroke lesion volume (ml)**
Internal/ext capsule/lentiform nucleus	Baseline	21	6.47 [3.43 9.31]	12.29 [6.63 23.46]	18 [11.70 31.54]	1.23 [0.99 1.77]	1.53 [1.12 3.66] (*n* = 12)
	1-year	16	6.82 [5.68 13.05]	19.77 [9.61 27.86]	27.41 [15.84 40.45]	0.49 [0.32 0.91]	1.37 [0.87 5.43] (*n* = 10)
Centrum semiovale	Baseline	33	9.62 [2.78 15.58]	24.04 [10.30 35.43]	33.69 [14.37 50.87]	1.32 [1.05 1.86]	2.25 [0.78 4.19] (*n* = 16)
	1-year	26	8.89 [3.01 16.82]	21.69 [12.34 30.28]	29.29 [15.95 47.78]	1.14 [0.75 1.46] (*n* = 25)	2.86 [0.89 3.61] (*n* = 11)
Thalamus	Baseline	13	2.44 [1.38 5.10]	4.52 [2.81 6.58]	6.89 [4.55 11.35]	0.51 [0.32 0.81]	0.26 [0.20 0.57] (*n* = 3)
	1-year	12	2.67 [1.77 4.52]	6.20 [2.57 10.65]	8.37 [5.02 15.17]	0.39 [0.19 0.53] (*n* = 10)	0.13 (*n* = 1)
Brain stem	Baseline	10	3.36 [2.02 10.94]	14.87 [7.81 29.75] (*n* = 9)	16.29 [8.43 43.0]	0.72 [0.56 0.98]	0.65 [0.42 0.76] (*n* = 5)
	1-year	6	3.14 [1.98 4.49]	14.37 [9.56 17.34]	17.51 [11.64 21.73]	0.14 [0.11 0.20] (*n* = 3)	0.48 [0.41 0.55] (*n* = 2)
Optical radiation	Baseline	2	6.05 [3.60 8.51]	27.17 [16.00 38.33]	33.22 [19.60 46.84]	1.60 [1.51 1.69]	0
	1-year	2	8.60 [4.79 12.41]	32.22 [18.76 45.69]	40.83 [23.55 58.10]	0.43 [0.36 0.50]	0

*Not all patients had previous (old) strokes. The acute lesion in some patients disappeared at 1-year. If the sample that contributed with non-zero data differs from the sample size in column 3 (from left to right), this is indicated in parenthesis*.

*RSSI location refers to RSSI lesion clusters in the anatomical locations specified. Three patients had RSSI lesion clusters DWI positive in more than one anatomical location*.

**Table 3:** Average (i.e., median) and interquartile range values [i.e., median (Q1 Q3)] of lesion volume (ml) per lacunes location in the baseline and follow-up samples.

**Lacunes location**	**Time point**	**Number of patients**	**Number of lacunes**	**Intense (severe) WMH volume (ml)**	**Less intense (subtle) WMH volume (ml)**	**Total WMH volume (ml)**	**Index stroke lesion (RSSI) volume (ml)**	**Old ischemic stroke lesion volume (ml)**
Internal/ext capsule/lentiform nucleus	Baseline	25	32	6.47 [2.44 9.70]	14.83 [6.43 27.21]	19.12 [9.91 39.96]	1.20 [0.76 2.10] (*n* = 22)	1.77 [0.81 4.18] (*n* = 21)
	1-year	20	27	5.81 [3.74 12.58]	15.51 [9.61 25.73]	20.71 [15.78 40.90]	0.76 [0.46 1.25] (*n* = 19)	1.93 [0.68 3.14] (*n* = 17)
Internal border zone	Baseline	2	2	6.70 [3.56 9.83]	19.97 [10.16 29.78]	26.66 [13.72 39.61]	0.11 (*n* = 1)	0.42 (*n* = 1)
	1-year	1	1	2.17	1.53	3.70	0	0
Centrum semiovale	Baseline	19	32	9.31 [6.33 17.51]	24.75 [14.51 30.20]	33.76 [18.82 45.26]	1.20 [0.98 1.59] (*n* = 18)	2.78 [1.27 4.33] (*n* = 18)
	1-year	12	20	11.81 [7.70 14.81]	21.77 [15.62 30.88]	34.68 [20.98 43.85]	0.82 [0.43 1.32]	2.86 [1.27 5.12]
Thalamus	Baseline	5	7	12.67 [9.53 19.81]	39.25 [24.75 47.13]	48.78 [36.13 67.51]	1.27 [0.96 2.18]	3.65 [2.66 4.23] (*n* = 4)
	1-year	4	5	21.13 [12.30 28.65]	29.18 [25.37 36.51]	50.31 [37.67 65.16]	1.62 [1.02 2.13]	2.86 [2.01 3.47] (*n* = 3)
Brain stem	Baseline	5	5	13.66 [9.31 15.58]	30.66 [29.0 35.29]	42.66 [39.96 50.87]	1.55 [0.88 2.34]	3.93 [0.52 6.48]
	1-year	4	4	15.46 [14.27 22.37]	32.19 [29.09 38.02]	45.90 [42.54 59.46]	1.41 [0.92 1.81]	5.27 [1.71 9.28]
Cerebellum	Baseline	3	3	2.44 [2.23 13.96]	3.44 [3.04 24.75]	5.88 [5.27 38.71]	0.72 [0.41 1.15]	0.81 [0.52 0.85]
	1-year	1	1	2.70	8.31	11.01	0.76	0.021
Optical radiation	Baseline	7	7	9.36 [5.71 10.33]	23.20 [18.00 29.83]	32.57 [23.51 41.31]	1.53 [0.99 2.20] (*n* = 6)	2.81 [1.78 6.48] (*n* = 5)
	1-year	7	7	11.03 [3.63 14.33]	23.99 [13.42 27.41]	29.29 [19.69 41.72]	0.41 [0.15 1.90] (*n* = 6)	5.77 [2.71 9.28] (*n* = 4)

*Not all patients had previous (old) strokes or identifiable RSSI (i.e., Index stroke) lesion. If the sample that contributed with non-zero data differs from the sample size in column 3 (from left to right), this is indicated in parenthesis*.

### RSSI and Baseline WMH Volume

RSSI volume was associated with WMH volume (B = 6.465, SE = 2.591, *P* = 0.0145, univariate linear regression). However, this association disappeared after adjusting for age, number of lacunes and vascular risk factors (B = 3.250, SE = 2.117, *P* = 0.129).

With regards to RSSI location, patients with RSSI in the centrum semiovale had higher baseline WMH volume [median (QR1 −QR3) = 33.69 (14.37 50.87) ml] than patients with the RSSI elsewhere (Tables 2, 4). Severe (i.e., intense) and subtle WMH volumes were both greater in this patient group compared with the rest (Tables 2, 4). Patients with the RSSI in the thalamus (*n* = 13) had the smallest median volumes of WMH [median (QR1 QR3) = 6.89 (4.55 11.35) ml] at baseline (Table 2).

**Table 4:** Results (*P* values) from the Kruskal-Wallis test to compare patients grouped by RSSI/lacunes anatomical location (including hemisphere), in relation to the following volumetric brain measurements at both time points: intense (i.e., overt) WMH (iWMH), less intense (i.e., subtle) WMH (LiWMH), index stroke lesion (Index SL) and old stroke lesion (Old SL).

**Lesion Location**	**Baseline**				**1-year follow-up**			
	**iWMH**	**LiWMH**	**Index SL**	**Old SL**	**iWMH**	**LiWMH**	**Index SL**	**Old SL**
**RSSI right hemisphere**								
Lentiform (*n* = 13)	0.285	0.874	0.497	0.723	0.159	0.265	0.321	0.922
CSO (*n* = 18)	0.044*	0.075	0.128	0.387	0.152	0.164	0.022*	0.855
**RSSI left hemisphere**								
Lentiform (*n* = 9)	0.325	0.692	0.117	0.324	0.492	0.901	0.366	0.887
CSO (*n* = 16)	0.008*	0.001*	0.145	0.353	0.055	0.011*	0.089	0.495
**Lacunes right hemisphere**								
Lentiform (*n* = 17)	0.349	0.735	0.204	0.842	0.592	0.521	0.486	0.830
CSO (*n* = 18)	0.022*	0.041*	0.514	0.013*	0.024*	0.172	0.701	0.207
**Lacunes left hemisphere**								
Lentiform (*n* = 15)	0.050	0.095	0.864	0.045*	0.014*	0.156	0.616	0.203
CSO (*n* = 14)	0.008*	0.012*	0.642	0.021*	0.055	0.111	0.229	0.080

*Significant difference were considered P < 0.05. Significant values are highlighted in bold and with an asterisk. “Lentiform” corresponds to RSSI/lacunes in the internal/external capsules/lentiform nucleus and “CSO” corresponds to RSSI/lacunes in the centrum semiovale. The number of patients with RSSI/lacunes in other locations was not enough to run statistical tests (see Table 1)*.

### Lacunes and Baseline WMH Volume

Number of lacunes was associated with WMH volume (B = 0.283, SE = 0.117, *P* = 0.0176, univariate linear regression). The association strengthened after adjusting for age, RSSI location, and vascular risk factors (B = 0.370, SE = 0.0939, *P* = 0.000174). Patients with lacunes had higher median WMH volumes than the median WMH volume in the (baseline) sample [median (QR1 QR3) = 14.37 (4.55 36.13) ml], with the exception of patients with lacunes in the cerebellum [*n* = 3 5.88 (5.27 38.71) ml].

With regards to lacunes location, although the number of patients with lacunes in the thalamus and brainstem was small, precluding statistical group comparisons, it is worth noting that patients with lacunes in these locations had the highest volumes of WMH in the sample. Patients with lacunes in the thalamus (*n* = 5) had the highest baseline WMH volume [median (QR1 QR3) = 48.78 (36.13 67.51) ml] followed by those with lacunes in the brainstem [42.66 (39.96 50.87) ml] (see Table 3).

### RSSI and WMH Volume Change

RSSI volume predicted the 1-year WMH volume change in this sample. In the ANCOVA model that also included age and number of lacunes as covariates but did not account for vascular risk factors, the estimate for RSSI volume was B = 3.238, standard error SE = 0.925, *P* = 0.000756. After accounting for vascular risk factors in the model, these values were B = 2.944, SE = 0.913, *P* = 0.00184.

**Figure 2:**
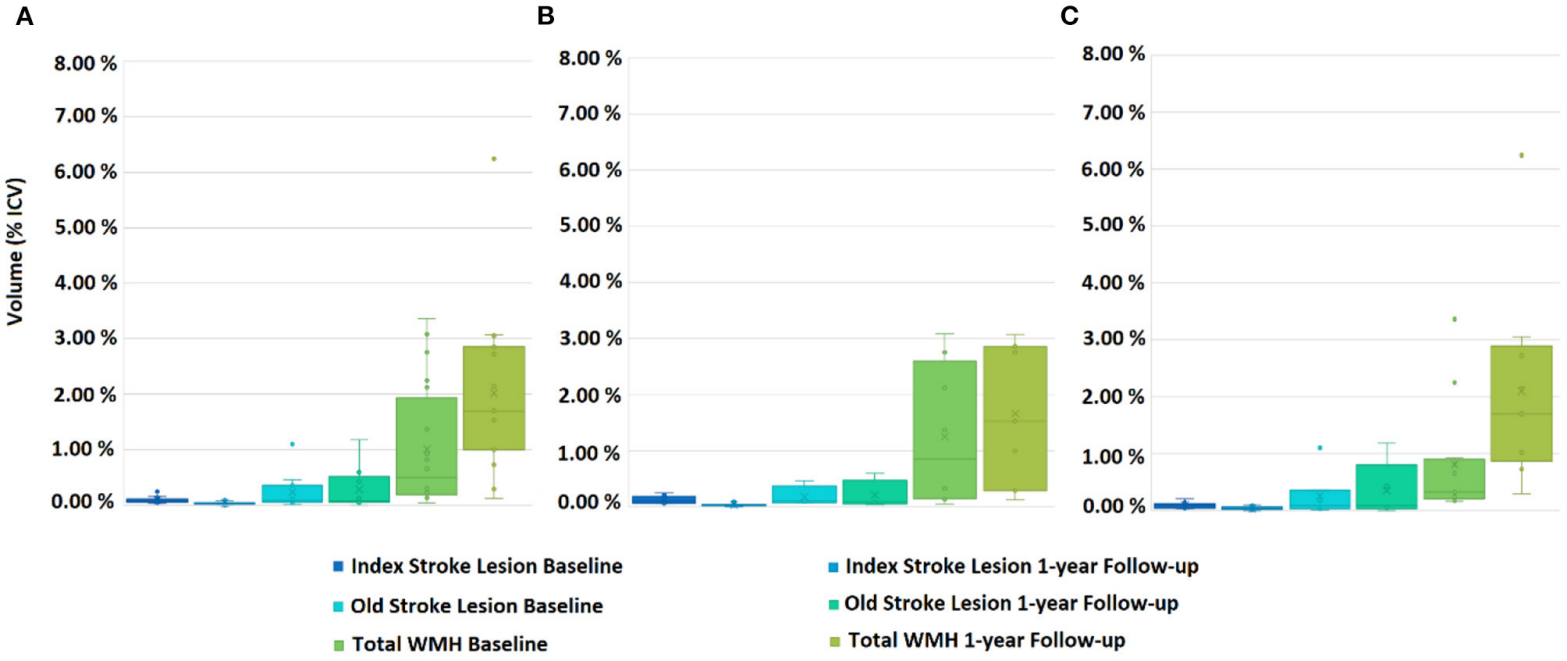
Box plots showing the distribution of the Total WMH (i.e., subtle/less intense and overt/intense combined together), acute and old stroke lesion volumes for patients with the RSSI at the internal/external capsule/lentiform nucleus, at baseline and 1-year follow-up. **(A)** Both hemispheres combined, **(B)** left hemisphere, and **(C)** right hemisphere.

**Figure 3:**
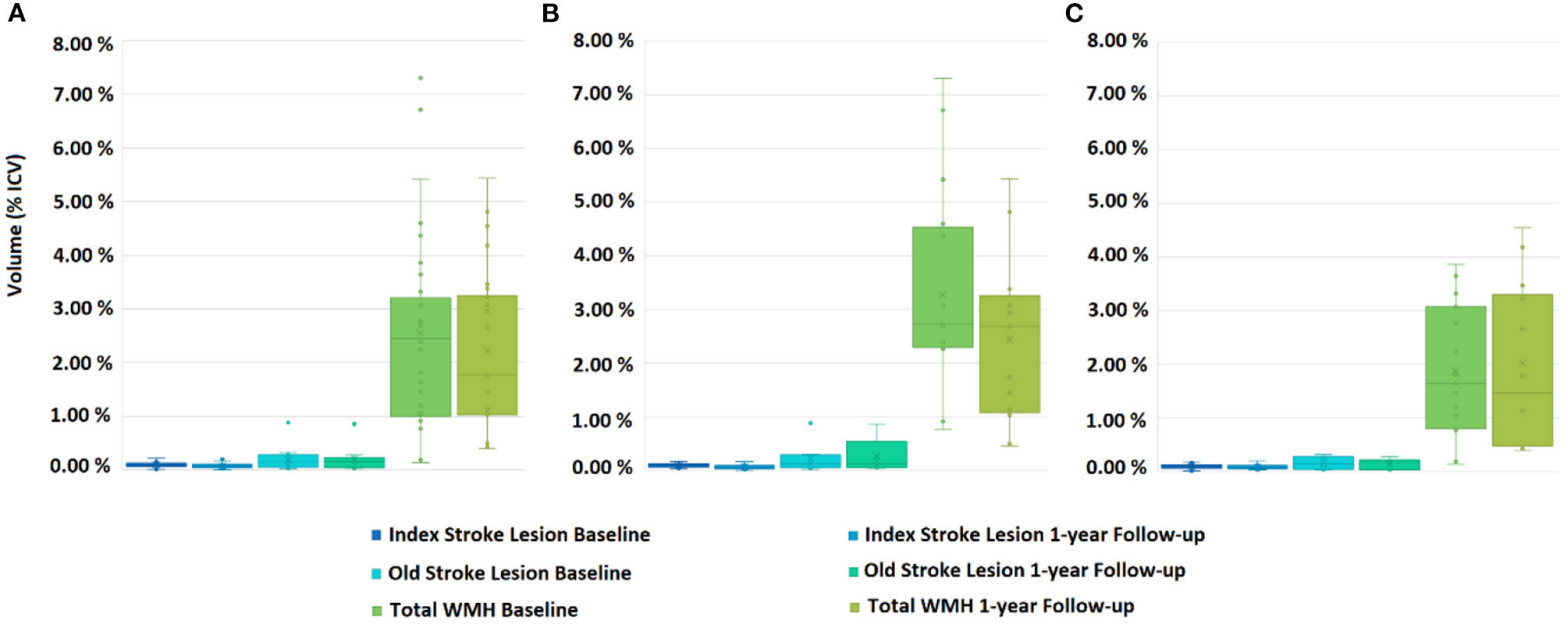
Box plots showing the distribution of Total WMH (i.e., subtle/less intense and overt/intense combined together), acute and old stroke lesion volumes for patients with the RSSI at the centrum semiovale, at baseline and 1-year follow-up. **(A)** Both hemispheres combined, **(B)** left hemisphere, and **(C)** right hemisphere.

RSSI location did not predict WMH volume change on any model (B = 0.00557, SE = 0.00388, *P* = 0.155 when accounting for age and number of lacunes; B = 0.00431, SE = 0.00381, *P* = 0.261 when also accounting for vascular risk factors, and B = 0.00609, SE = 0.00388, *P* = 0.121 when accounting for age, number of lacunes and volume of previous stroke lesions). Figures 2, 3 show the lesion (i.e., RSSI, old ischemic strokes and WMH) change for patients with the RSSI in the internal/external capsule/lentiform nucleus and centrum semiovale, respectively, after these being adjusted for head size. The median WMH volume in patients with the RSSI in the centrum semiovale (43% of the sample) was smaller at 1-year [median (QR1 QR3) = 29.29 (15.95 47.78) ml] than at baseline [33.69 (14.37 50.87) ml], but this difference did not reach statistical significance. The median WMH volume for patients with the RSSI elsewhere slightly increased at 1-year (Table 2). Within patients with the RSSI in the internal/external capsule/lentiform nucleus, the 1-year increase in WMH volume was particularly noticeable for those with the RSSI in the right hemisphere (Figures 2B,C, Tables 2, 4).

### Lacunes and WMH Volume Change

Number of lacunes did not predict WMH volume change. Supplementary Table 1 shows the median [QR1 QR3] lesion volume change in patients grouped by lacunes location and in the total sample. Patients with lacunes in the thalamus not only had the highest volumes of WMH at baseline (i.e., mentioned in previous section), but also the highest increase of these values 1 year after the stroke [median change (QR1 QR3) = 10.77 (−1.62 18.90) ml (*n* = 4)], while patients with lacunes in the brainstem (*n* = 7) had the highest reduction [median change (QR1 QR3) = −1.16 (−7.23 0.26) ml] (see Supplementary Table 1).

### Voxel-Based Analysis

**Figure 4:**
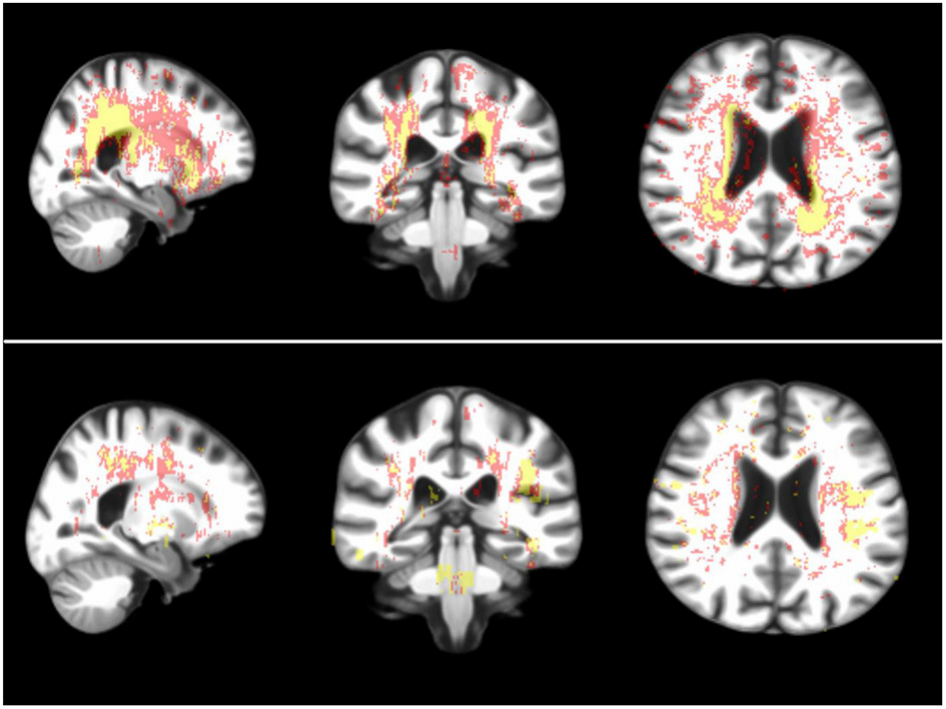
Sagittal, coronal and axial representative views (from left to right) of brain regions where the WMH of the hypertensive (above) and smoker (i.e., recent and current combined, below) patients with RSSI in the centrum semiovale statistically significantly differed from the rest. In red: voxels where the group comparison (i.e., Kruskal-Wallis test) resulted in *P* < 0.05 after correction for multiple comparisons (i.e., false discovery rate) and in yellow: voxels where *P* < 0.01.

Baseline WMH in patients with the RSSI or lacunes in the external capsule/lentiform nucleus did not differ from baseline WMH in patients with the RSSI or lacunes elsewhere. However, baseline WMH in the subset of current and recent smokers within this group significantly differed from the rest in wide clusters across the whole brain (*P* < 0.01). Table 5 shows the number of baseline WMH voxels that significantly differed between patients grouped per RSSI or lacunes location and with different vascular risk factors after correction for multiple comparisons. Baseline WMH in patients with the RSSI or lacunes in the centrum semiovale differed from the rest in more locations, especially in hypertensive patients (Figure 4, Table 5).

**Table 5:** Results from the voxel-based group comparisons (Kruskal-Wallis test) of the baseline WMH maps (i.e., considering both overt/intense and subtle/less intense together) of patients grouped by RSSI or lacunes location and risk factors, after correcting for multiple comparisons.

**Patient group**	**No. of voxels** **(WMH volume in ml) statistically significantly different at cut-off** ***P* < 0.05**	**No. of voxels** **(WMH volume in ml) statistically significantly different at cut-off** ***P* < 0.01**
**RSSI in internal/external capsule/lentiform nucleus**	0 (0)	0 (0)
RSSI in internal/external capsule/lentiform nucleus hypertensive	893 (0.89)	150 (0.15)
RSSI in internal/external capsule/lentiform nucleus with hypercholesterolemia	2,251 (2.25)	80 (0.80)
RSSI in internal/external capsule/lentiform nucleus current and recent smokers	14,253 (14.25)	5,530 (5.53)
**Lacunes in internal/external capsule/lentiform nucleus**	0 (0)	0 (0)
Lacunes in internal/external capsule/lentiform nucleus hypertensive	1,574 (1.57)	1 (0.001)
Lacunes in internal/external capsule/lentiform nucleus with hypercholesterolemia	2,197 (2.20)	2 (0.002)
Lacunes in internal/external capsule/lentiform nucleus current and recent smokers	27,477 (27.48)	9,536 (9.54)
**RSSI in centrum semiovale**	148,639 (148.64)	32,585 (32.58)
RSSI in centrum semiovale hypertensive	126,970 (126.97)	33,456 (33.46)
RSSI in centrum semiovale with hypercholesterolemia	8,317 (8.32)	0 (0)
RSSI in centrum semiovale current and recent smokers	61,664 (61.66)	28,112 (28.11)
**Lacunes in centrum semiovale**	53,233 (53.23)	13,601 (13.60)
Lacunes in centrum semiovale hypertensive	81,710 (81.71)	12,035 (12.04)
Lacunes in centrum semiovale with hypercholesterolemia	64,946 (64.95)	8,801 (8.80)
Lacunes in centrum semiovale current and recent smokers	37,098 (37.10)	24,084 (24.08)

*The number of RSSI/lacunes in other locations was not enough to run statistical tests (see Table 1)*.

**Figure 5:**
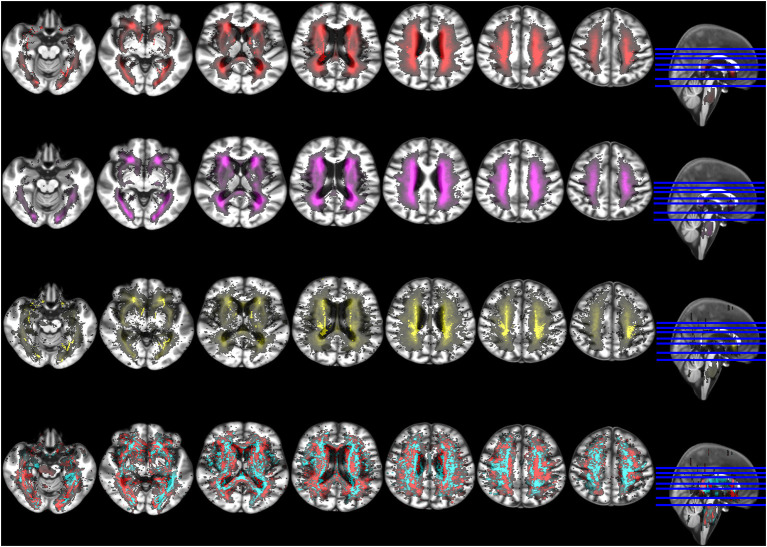
Selection of axial slices showing the probability distribution maps of WMH at baseline (upper row, red), WMH at 1-year follow-up (second row from the top, magenta), WMH change (second row from the bottom, yellow), and voxel-based associations between WMH change and RSSI location of the primary (i.e., larger) DWI positive cluster accounting for age (bottom row). In the latter, positive associations are shown in cyan and negative associations in red. Although positive associations were stronger than negatives in absolute values, for visual representation both maps were rescaled to the 50% of their maximum values.

**Figure 6:**
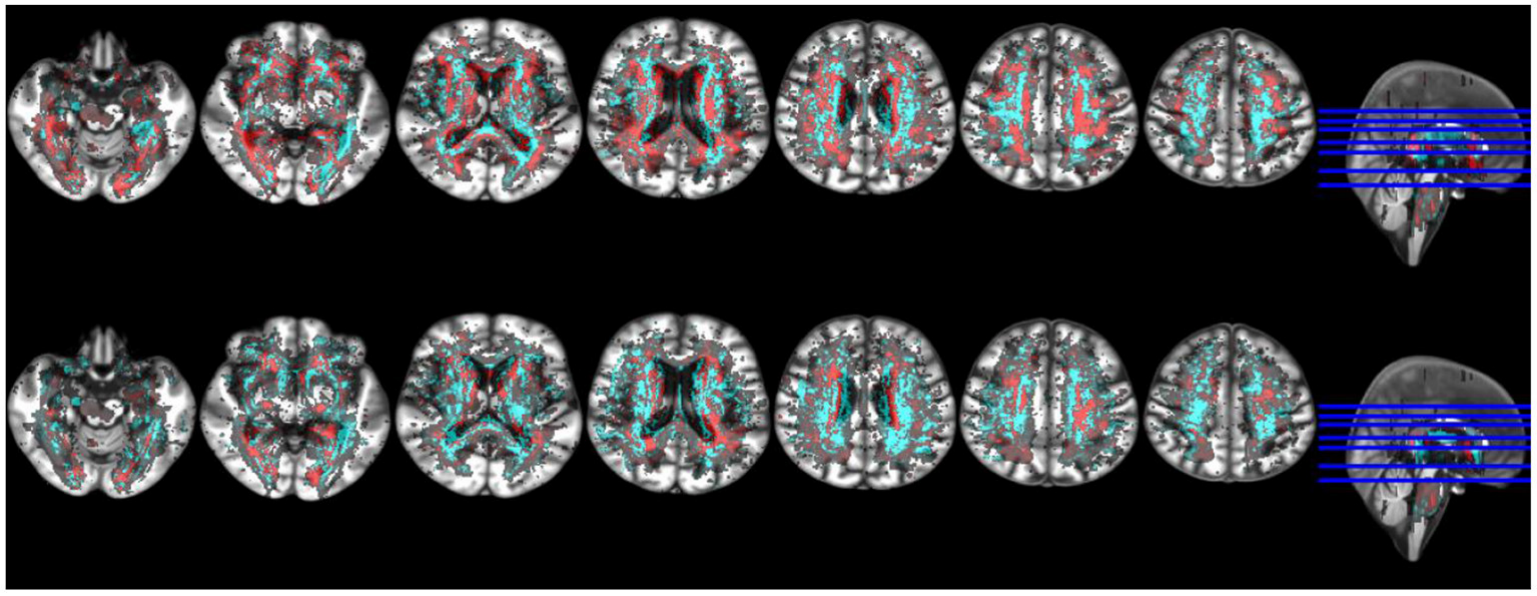
Selection of axial slices showing the probability distribution maps of the associations of WMH change with RSSI location of the primary (i.e., larger) DWI positive cluster accounting for age (top row) and RSSI location with baseline WMH (bottom row). In both, positive associations are shown in cyan and negative associations in red. Although positive associations were stronger than negatives in absolute values, maps were rescaled to the 50% of their maximum values to facilitate visual comparability.

RSSI location was more strongly associated with WMH evolution mainly at the regions of intersection between white matter tracts, although positive associations were seen in all major white matter pathways at the internal/external capsule, corpus callossum, and optical radiation. Negative associations were observed along the periventricular area, related to ventricular shape changes and ventricular enlargement, deep gray matter and corona radiata supraventricular (Figure 5). This pattern of associations differed from the one found between RSSI location and WMH burden at baseline (Figure 6).

## Discussion

In-line with our hypotheses, in our sample of patients presented to a hospital with an acute lacunar ischemic stroke clinical syndrome, WMH volume and distribution distinctively differed per RSSI and lacunes location, being this difference accentuated, in general, in the presence of vascular risk factors. Number of lacunes was associated with baseline WMH volume but did not predict its change. As such, patients with lacunes had higher baseline WMH volume than patients who did not have lacunes. RSSI volume was not associated with WMH volume but predicted its change 1 year post-stroke. RSSI location did not predict WMH volume change but influenced the pattern and 1-year evolution of its distribution. Being hypertensive or recent or current smoker accentuated the extent of differences in WMH distribution between patients grouped by RSSI location. We discuss each result in a separate subsection.

### RSSI and Lacunes Location and WMH Spatial Distribution

WMH spatial distribution in patients with the RSSI and lacunes in the centrum semiovale differed from the rest of the patient groups. However, WMH distribution in smokers and hypertensive patients with the RSSI and lacunes in the internal/external capsule/lentiform nucleus distinctively differed, independently, from WMH spatial distribution in patients with the SSI elsewhere in several locations. Smoking, in general, was influential of the WMH spatial distribution patterns per lesion location. Previous studies have shown smoking to be a relevant risk factor for increased WMH volume (32), loss of white matter integrity (33), and SVD progression (34). Our results reinforce the relevance of smoking to brain health, informing on specific locations that seem to be more vulnerable than others depending on the location of the lacunar infarct. If replicated in wider samples, could be informative for prognosis and recovery after stroke.

### RSSI Location and WMH Volume and Progression

WMH volume significantly increased in the 1-year period for patients with the RSSI in the internal/external capsule/lentiform nucleus, especially for those with the RSSI in the right hemisphere. However, the WMH volume in patients with the RSSI in the centrum semiovale was significantly higher than the WMH volume in the internal/external capsule/lentiform nucleus at baseline. Although our results may imply that the SVD dynamics (10, 35, 36) and short-term (i.e., 1-year) outcome differ by location of the subcortical infarct, it is worth noting that the majority of patients with the RSSI in the centrum semiovale were hypertensive (i.e., 81.8%). Hypertension has been associated with WMH volume (13, 21, 37) in statistical models adjusted by age. Therefore, it is not surprising that the baseline WMH volume in this patient group is, overall, higher than the WMH volume in the rest of the patients with the RSSI located elsewhere, which had fewer proportion of hypertensive patients. It is worth noting that patients with the RSSI in the thalamus had the smallest WMH volume at baseline and its higher increase at 1 year. However, the small number of patients with the RSSI in this location precludes us from drawing conclusions.

### Influence of RSSI Location in WMH Evolution

In our sample, WMH evolution at 1 year was associated to RSSI location in distinctive clusters distributed across the brain, in some of which the baseline association of WMH and RSSI location had opposite direction. None of the patients had any symptomatic event that required an additional MRI examination in the time between these two time-points. The majority of lacunar strokes are acknowledged to be asymptomatic (19), presenting symptoms mainly those that occur in the vicinities (or in the path of) the sensory and motor white matter tracts (4). It is possible that some of the hyperintensities classed as WMH at follow-up could be silent subcortical infarcts (38, 39). Given the dynamic nature of both of these features, and SVD as a whole (10, 35, 36), without frequent MRI examination (e.g., monthly, bi-monthly, quarterly) it is impossible to determine the cause-effect relation between RSSI location and WMH evolution. However, our results, if confirmed in larger samples, indicate that RSSI location and stroke symptomatology may be predictors of the evolutionary pattern of white matter disease a year post-stroke, and, therefore, combined, could be further evaluated for their use in precision medicine to develop personalized therapeutic approaches.

### Interhemispheric Differences in Baseline WMH Volumes

In our sample, the majority of the acute infarcts occurred in the centrum semiovale. In these patients the baseline WMH volume in the left hemisphere was significantly higher than in the right hemisphere. Our results complement those from Ryu et al. (19), who suggested that patients with asymmetric WMH may be vulnerable to lacunar infarct, especially those with old silent lacunar infarcts, in a sample with the RSSI predominantly in the corona radiata (compared to the striatocapsular region).

The second location with most acute infarcts was the internal/external capsule/lentiform nucleus. In this patient group the baseline WMH volume between both hemispheres did not significantly differ, although a higher inter-individual variability was observed in the left hemisphere. These results are in-line with those from a cross-sectional study on a larger sample (*n* = 187) of symptomatic DWI-positive RSSI, mainly located at the internal/external capsule/lentiform nucleus (4), who reported no statistically significant inter-hemispheric differences in WMH volumes (40). The same study also reported high inter-individual variability on the total WMH volumes depending on RSSI hemisphere.

### Strengths and Limitations

Although this sample was moderate-to-large in regard to lacunar strokes, less patients attended the follow-up assessment. The unbalanced number of patients regarding RSSI and lacunes location, with few patients (or none) presenting with the RSSI in certain locations, limited the analyses and biases some of our results toward the locations where most of the acute infarcts occurred, i.e., the centrum semiovale, followed by the internal/external capsule/lentiform nucleus. The inclusion of the few patients with more than one RSSI lesion cluster in the analysis is another limitation of the study, as the cause of stroke may differ from the rest. However, to exclude them would have introduced a selection bias not justified by the clinical data available. Another limitation of our study is the lack of MRI data in the 1-year interval (i.e., intermediate, short-term follow-up examinations between baseline and 1-year scans), which precludes us from analyzing the cause-effect interaction between SSI symptomatology (i.e., silent vs. not-silent), SSI location and WMH.

Among the strengths of our study are the longitudinal analysis of MRI acquired following the same protocol in the same research scanner, the neuroradiological assessment using well-established guidelines, the availability of relevant clinical data, as well as the application of state-of-the-art imaging processing and analysis methods, made publicly available. Repeating our models accounting for the location of multiple DWI-positive clusters did not change the overall results of the analyses. Our results add to the current body of knowledge on lacunar stroke and SVD, with possible implications for predicting stroke risk and short-term outcome if they are replicated in wider samples.

## Data Availability Statement

Data is available upon request or accessible via Meta VCI Consortium: https://metavcimap.org/group/consortium/.

## Ethics Statement

The studies involving human participants were reviewed and approved by Lothian Ethics of Medical Research Committee (REC 09/81101/54) and NHS Lothian R+D Office (2009/W/NEU/14), on the 29th of October 2009. The patients/participants provided their written informed consent to participate in this study.

## Author Contributions

MV: study design, image processing, data generation, data analysis, and writing the manuscript. TG-M: data analysis and writing the manuscript. FC: statistical analysis oversight, revising, and editing the manuscript. SM: data collection, patient recruitment, clinical examination, revising, and editing the manuscript. MT and PA: image protocol and acquisition oversight, revising, and editing the manuscript. ES: image processing, data generation, revising, and editing the manuscript. JW: study design, image analysis, data generation, revising, and editing the manuscript. All authors: contributed to the article and approved the submitted version.

## Conflict of Interest

The authors declare that the research was conducted in the absence of any commercial or financial relationships that could be construed as a potential conflict of interest.
