# Supplementary material for: Lacunar Stroke Lesion Extent and Location and White Matter Hyperintensities Evolution 1 Year Post-lacunar Stroke
Source: Front Neurol. 2021 Mar 5;12:640498. doi: 10.3389/fneur.2021.640498 (PMC7976454; doi:10.3389/fneur.2021.640498)

| Lacunes location | Number of patients | Intense (severe) WMH volume change (ml) | Less intense (subtle) WMH volume change (ml) | Total WMH volume change (ml) | Index stroke lesion (RSSI) volume change (ml) | Old ischaemic stroke lesion volume change (ml) |
| --- | --- | --- | --- | --- | --- | --- |
| Internal/ext capsule / lentiform nucleus | 20 | 0.16 [-1.18 2.07] | 1.63 [-0.93 4.24] | 1.42 [-0.98 3.40] | -0.36 [-0.76 0.031] (n=19) | -0.15 [-0.35 0.15] (n=17) |
| Internal Border Zone | 1 | 1.75 | 1.18 | 2.93 | 0 | 0 |
| Centrum Semiovale | 12 | 1.47 [-0.29 3.85] | 0.98 [-1.79 4.19] | 1.15 [-0.51 2.97] | -0.23 [-0.79 0.15] | -0.05 [-0.64 0.20] |
| Thalamus | 4 | 6.44 [0.42 13.62] | 4.33 [-2.04 5.28] | 10.77 [-1.62 18.90] | -0.10 [-0.35 0.13] | -0.091 [-0.71 0.43] (n=3) |
| Brain Stem | 4 | 1.51 [-0.83 4.52] | -4.18 [-11.35 -0.82] | -1.16 [-7.23 0.26] | -0.57 [-0.96 -0.26] | -0.19 [-0.68 0.43] |
| Cerebellum | 1 | 0.68 | 5.68 | 6.36 | 0.038 | -0.21 |
| Optical Radiation | 7 | 0.28 [-0.55 3.46] | -3.54 [-5.81 2.91] | -0.52 [-3.41 0.41] | -0.76 [-1.11 -0.16] (n=6) | -0.31 [-0.35 0.34] (n=4) |
| Total sample | 88 | 0.51 [-0.63 1.72] (n=87) | 0.87 [-2.46 3.58] (n=87) | 1.30 [-2.23 3.85] | -0.43 [-0.77 -0.068] (n=56) | -0.13 [-0.35 0.15] (n=26) |

Supplementary Table 1. Median and interquartile range values (i.e., shown as median [Interquartile range]) of the lesion volume change (expressed in ml) per lacunes location in the subsample that had follow-up. Not all patients had previous (old) strokes or identifiable RSSI lesion. If the sample that contributed with non-zero data differs from the sample size in column 2 (from left to right), this is indicated in parenthesis.


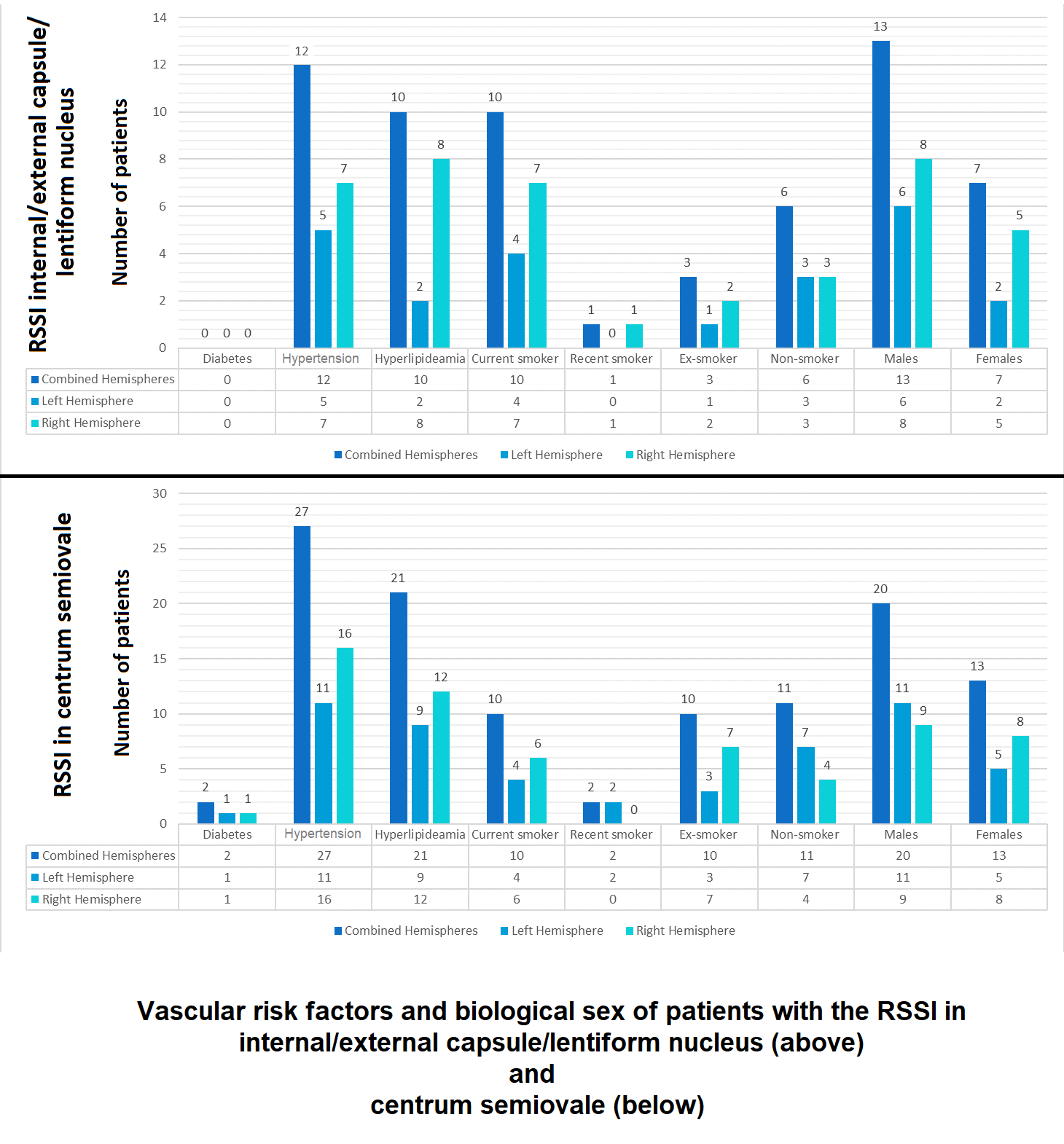


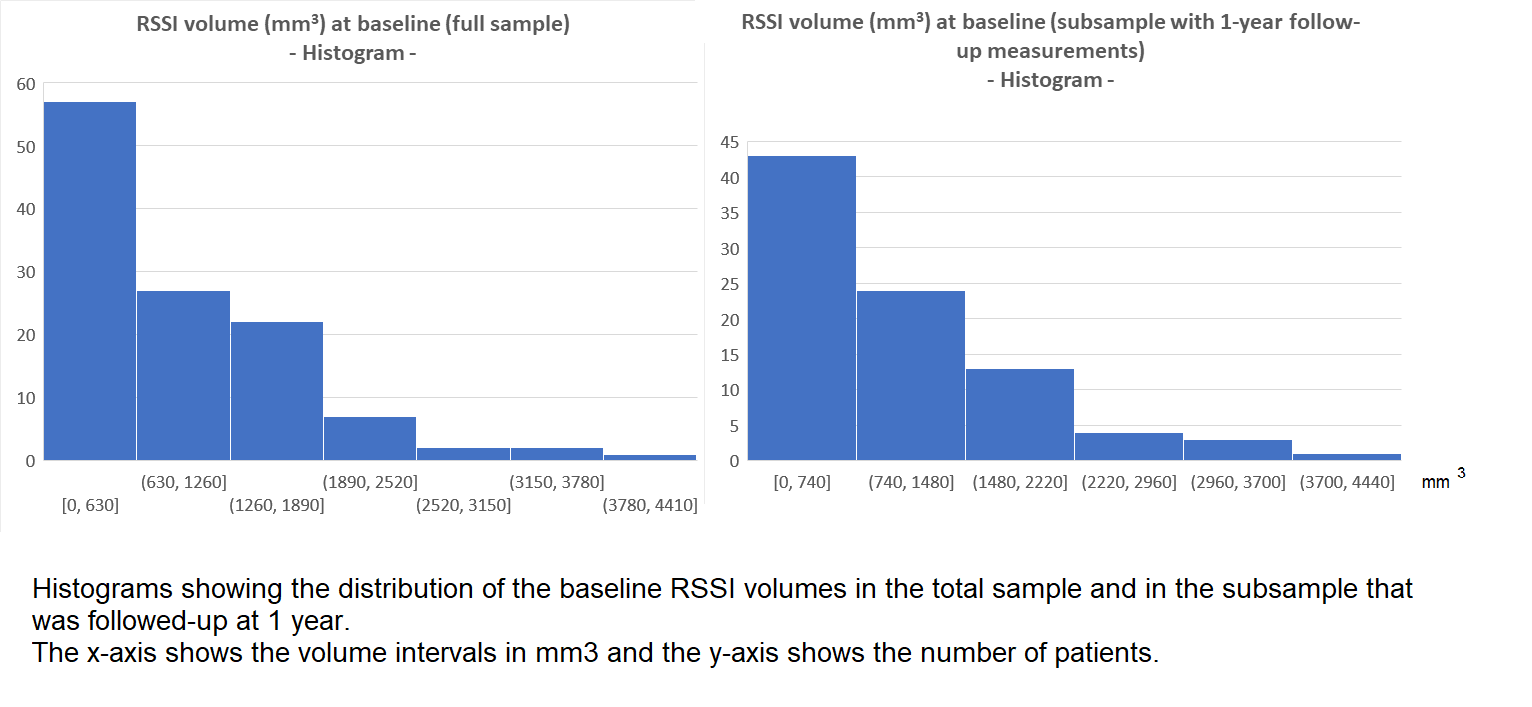

Supplement: Supplementary file 1 [file Data_Sheet_1.docx]
